# Accuracy of Percutaneous CT-Guided Spine Biopsy and Determinants of Biopsy Success

**DOI:** 10.5334/jbr-btr.985

**Published:** 2016-05-31

**Authors:** Selim Baris Gul, Ahmet Veysel Polat, Tumay Bekci, Mustafa Bekir Selcuk

**Affiliations:** 1 Aksaray State Hospital, TR; 2 Ondokuz Mayis University Faculty of Medicine, TR

**Keywords:** Computed tomography, Spine, Needle biopsy, Vertebral lesions

## Abstract

**Purpose:**

The purpose of this study was to investigate the accuracy of CT-guided spine biopsy as well as the factors that may influence its success.

**Methods and Materials:**

A total of 170 CT-guided biopsies performed on 156 patients with vertebral lesions were retrospectively analyzed. The accuracy of the biopsies was evaluated by comparing the final diagnosis with the biopsy results for patients who underwent surgery or with six-month clinical and radiological follow-up findings for patients who did not have surgery. The radiological features of each lesion, the features of the needles used, the needle approach, the pathology results, and the patient demographic data were statistically analyzed with Fisher exact test and ANOVA for their influence on the success of the biopsy.

**Results:**

The total success rate of percutaneous vertebral needle biopsies performed with CT guidance was 80 per cent (136/170). Age (*p* = 0.39), gender (*p* = 0.43), lesion location (*p* = 0.2), radiographic appearance (*p* = 0.8), needle type (*p* = 0.6), and approach (*p* = 0.1) had no effect on the adequacy of the obtained material or the success of the biopsy. There was a relationship between lesion histopathology and the rate of adequacy or success (*p* < 0.001). There was no relationship between the needle approach or the radiographic appearance of the lesion and the length of the specimen (*p* = 0.1). There were no major complications requiring treatment.

**Conclusion:**

The success rate of CT-guided percutaneous needle biopsy was close to that found in previous studies in the literature and independent of most patient parameters. Its complication rates are acceptable in experienced hands.

## Introduction

The most important step in the process of diagnosing a neoplastic lesion, or excluding a probable malignancy, is performing a biopsy that can provide an accurate diagnosis. The traditional method of open biopsy, which gathers sufficient material for histological and immunohistochemical studies in musculoskeletal system lesions, has been considered the gold standard. However, since open biopsy of vertebral lesions is difficult, the risk of complications is high [1,2,3,4,5,6,7,8,9]. As an alternative to open biopsy, percutaneous needle biopsy, which has a higher accuracy rate and is less invasive, has recently become more popular [10,11,12,13,14,15]. Percutaneous needle biopsy can be performed on an outpatient basis and usually does not require general anesthesia. Its cost and duration are lower compared to open biopsy, and there is a lower risk of metastasis, infection, and wound-site complications. In addition, percutaneous needle biopsy is preferred to other procedures among the musculoskeletal system interventional methods, especially in the presence of deep lesions of the pelvis or vertebrae.

The aim of this study was to determine the diagnostic accuracy of the CT-guided vertebral lesion biopsy and to investigate possible factors that may affect its success, such as age, gender, radiologic appearance, lesion localization and size, needle type, and needle approach. For this purpose, we reviewed the results of CT-guided percutaneous needle biopsies performed in our interventional radiology unit over a period of six years.

## Material and Methods

### Patients

The results of 187 percutaneous biopsies of vertebral lesions performed at Ondokuz Mayıs University Faculty of Medicine, Department of Radiology, between February 2009 and October 2015, were reviewed retrospectively, along with the patient data. Informed consent about the procedure was obtained from the patients before the interventions. The hospital’s ethics committee approved this retrospective study.

The data included patient demographics, biopsy technique, imaging technique, lesion histopathology, and clinical and radiological follow-up findings. Percutaneous biopsy pathology results were compared with open biopsy pathology results for patients who underwent surgery. For the patients who did not undergo surgery, percutaneous needle biopsy results were compared with six-month clinical or radiological follow-up findings and with the final clinical diagnosis. Seventeen cases that did not meet the six-month clinical follow-up criteria were excluded. The remaining 170 biopsies (156 patients) were used for statistical analysis. Eighty-seven of the patients were male, and 69 were female, with an average age of 56 years (range 8–86 years).

### Procedure

Radiography, CT, MRI, and bone scintigraphy were performed to evaluate the spread of lesions and for a definitive diagnosis. In the presence of multiple lesions, the most easily accessible biopsy site was chosen. Local anesthesia was used for all patients, with a 5–10 cc injection of prilocaine hydrochloride. For children and older patients who had difficulty lying on the CT table for reasons such as pain, sedation was provided.

Each biopsy was performed with either a Jamshidi needle (Somatex, Berlin, Germany) or a Tru-Cut needle (Geotek, Ankara, Turkey). The Jamshidi needle, used in 137 biopsies, was preferred in the presence of an intact cortex, while the Tru-Cut needle, used in 30 biopsies, was preferred in the presence of a very thin cortex or soft tissue not covered by bone. Fine needle aspiration biopsy (FNAB) was performed on only three patients. There were only two cervical-level cases in our patient group, and since both had posterior lesions, their interventions required the posterior approach. For the thoracic and lumbar spine, biopsies were performed in the prone position for all other cases.

For each procedure, a scanogram was obtained with CT (CT Sytec Plus, GE). Next, CT sections were obtained of the lesion-containing vertebrae. The slice thickness was set at ≤5 mm, depending on vertebral level and lesion size. The most suitable point of entrance was identified on the CT images and marked on the skin with a pen. Local anesthesia was administered after the intervention area was sterilized. The biopsy needle was inserted, and CT images were repeated at the same level to confirm the correct needle direction. If the needle was not correctly directed at the lesion, its angle was adjusted, moving the needle slowly to prevent false negative results or complications. The biopsy specimens were sent to the pathology laboratory in gauze dressings soaked with physiological saline solution when the sample included bone or in formol-filled tubes when only soft tissues were obtained.

All of the specimens were checked for sufficiency. The needle biopsy results for those with sufficient samples were compared to the corresponding final diagnoses. Needle biopsies that were compatible with the histopathology results or the final diagnosis after six months of follow-up (in the open-biopsy cases) were considered successful. Biopsies incompatible with the final diagnosis and those deemed insufficient for analysis were considered unsuccessful. The sufficiency of the material and the accuracy of the biopsy were assessed with regard to age; gender; anatomic level of the lesion (cervical, thoracic, lumbar, or sacral); lesion size; histopathological results (primary malignancy, secondary malignancy, or inflammation/infection); needle type (Jamshidi, cutting needle, or thin needle); needle diameter (11-, 13-, 14-, or 20-gauge); radiological appearance of the lesion (lytic, sclerotic, or mixed); presence or absence of a primary malignancy; and approach (transcostovertebral, posterior, posterolateral, or transpedicular). The average length of biopsy samples obtained with different needles and the average length of samples from lesions with different radiological appearances were assessed, and the association between sample size and sufficiency was calculated.

### Statistical analysis

Statistical analyses were performed with SPSS version 15.0, and qualitative data were compared with the chi-square test. Fisher’s exact test was used to compare the biopsy success rate between genders, and ANOVA was used to determine whether there was an association between specimen size and the type of needle used or the radiological appearance of the lesion. A *p* value of <0.05 was considered statistically significant.

## Results

When the results of 170 biopsies performed on 156 patients were analyzed, 152 (89.4%) were found to involve sufficient specimens. Eighteen biopsies (10.6%) involved insufficient specimens, and therefore had no results. These biopsies were included in the unsuccessful biopsy group.

The accuracy rate of the 152 biopsies with sufficient material was 89.5 per cent (136/152). Sixteen biopsies (10.5%) were not accurate compared to the final diagnosis. The total success rate of percutaneous vertebral needle biopsies performed with CT guidance was 80 per cent (136/170). When the results were divided into successful and unsuccessful groups, no significant differences were found between the biopsies in terms of age (*p* = 0.39) or gender (*p* = 0.43). No statistically significant difference was found between the sufficiency of the obtained material and the success of the biopsy using different needle approaches, or between biopsy success and lesion size (greatest length measured at the anteroposterior, craniocaudal, or transverse level) or sufficiency of the obtained material.

**Figure 1:**
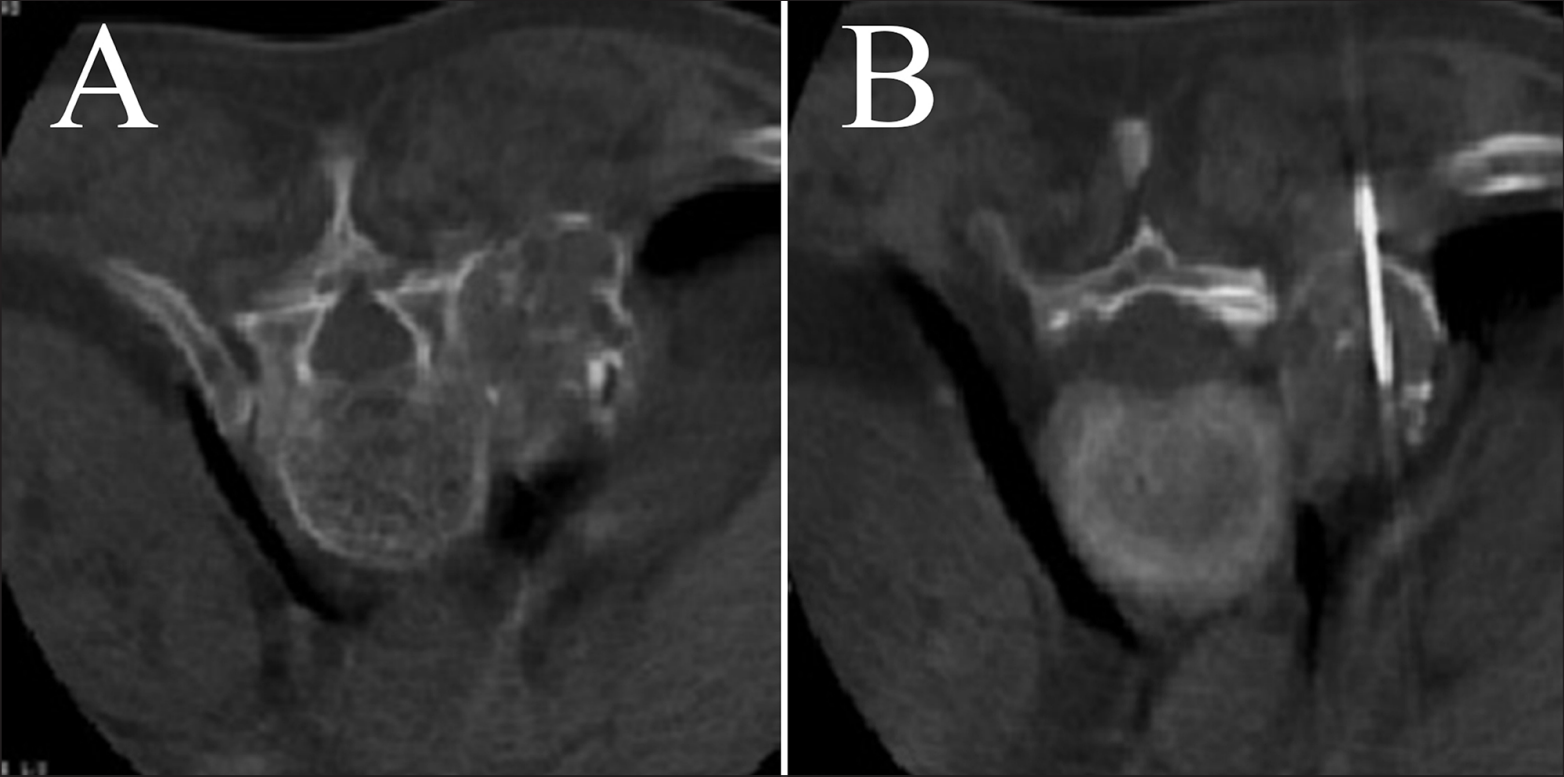
Exophytic lesion of the T11 vertebral pedicle in a 76-year-old female. A Jamshidi needle biopsy using a posterolateral approach was performed. The histopathology showed chondrosarcoma.

**Figure 2:**
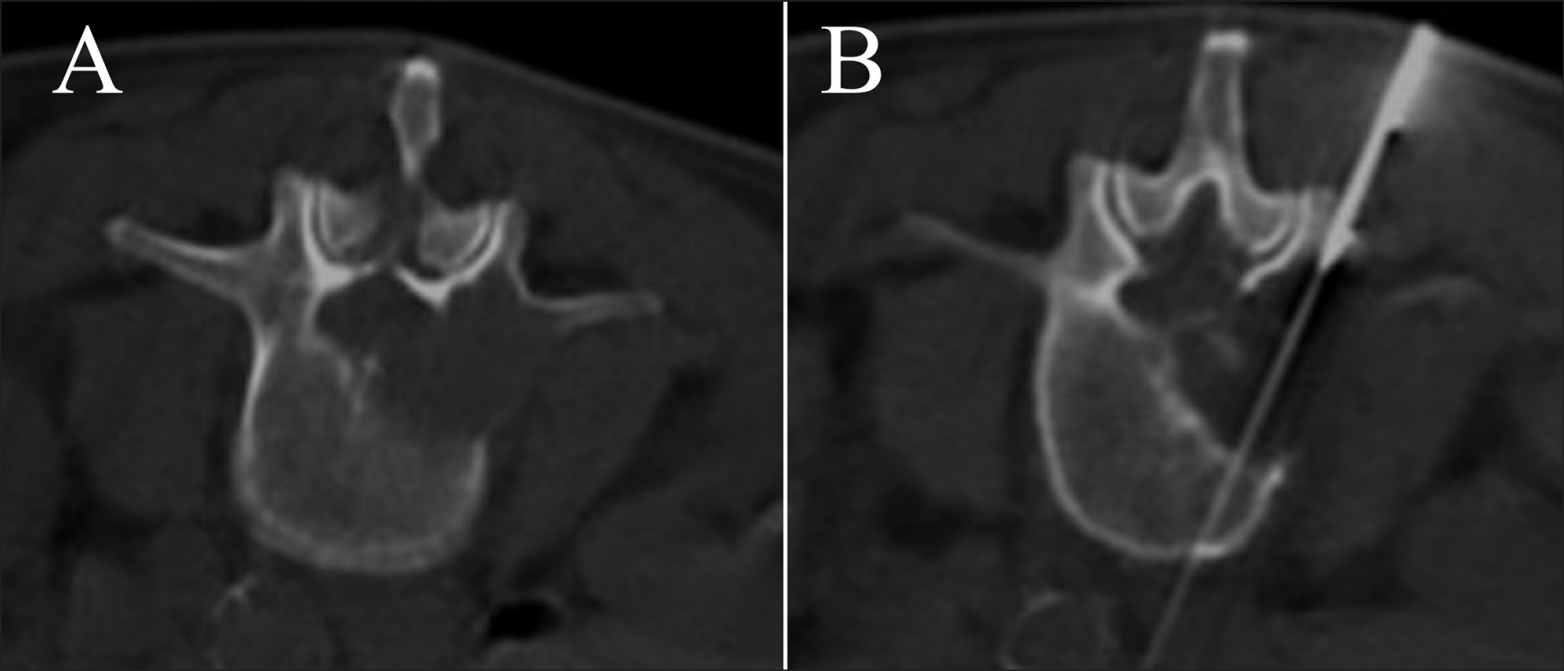
Lytic lesion involving the right pedicle of the L3 vertebra in a 73-year-old male. A Tru-Cut biopsy with a transpedicular approach was performed, and the histopathology showed metastatic carcinoma.

**Figure 3:**
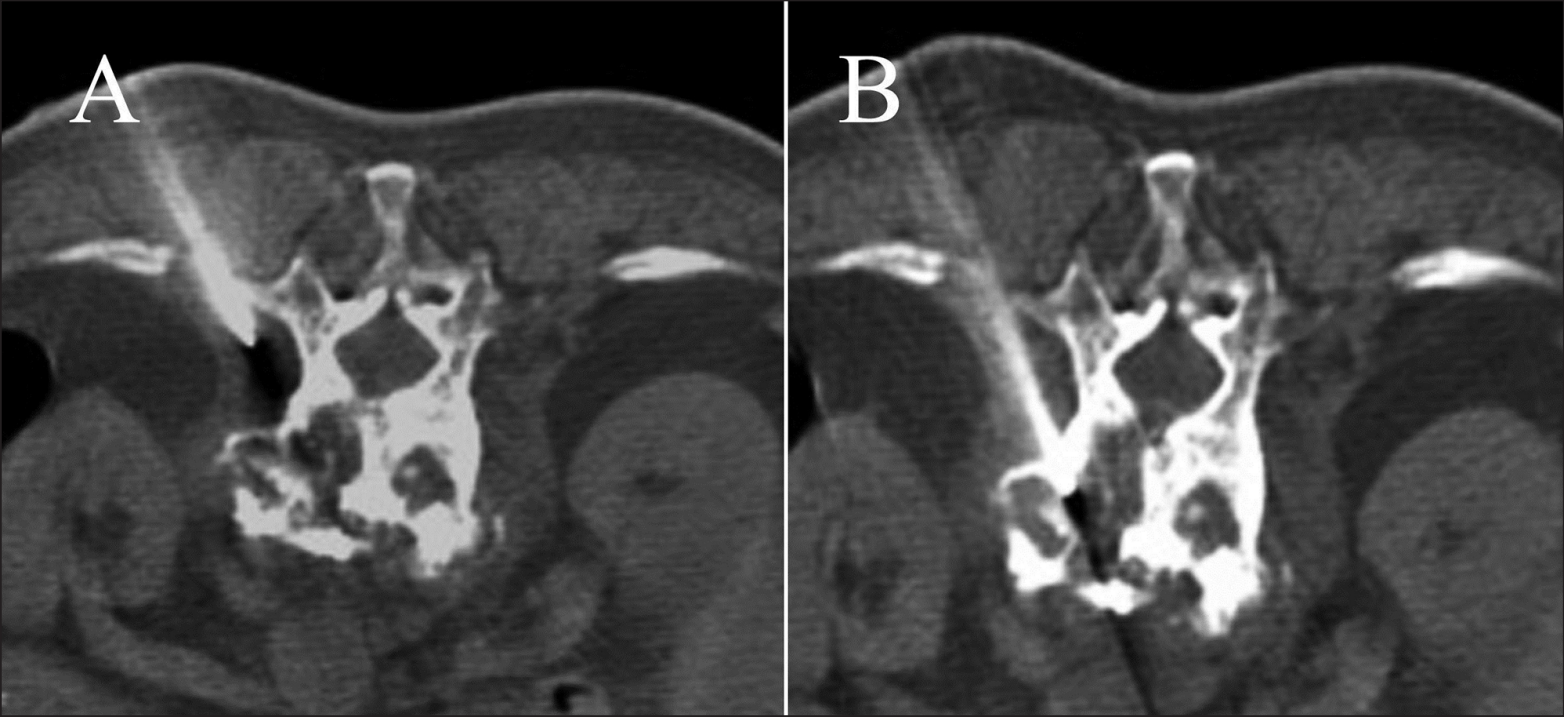
A 78-year-old female with a compression fracture of the T12 vertebra. A biopsy was performed using a transcostovertebral approach, and the histopathology showed reactive bone changes from a benign compression fracture.

The lesions were grouped according to histopathology as follows: primary malignant tumors (Figure 1), secondary malignant lesions (including metastases) (Figure 2), non-neoplastic infections with inflammatory changes or specific/nonspecific infections, and “other”. Biopsies that showed degenerated bone or cartilage tissue, hypo- or hypercellular bone marrow, osteoporosis, edema, or no specific pathology and different reactivity (Figure 3) were included in the “other” group. The relationship between histopathological groups, material sufficiency, and biopsy success is summarized in Table 1. No association was found between the radiological appearance of lesions and sufficient biopsy material or biopsy success (*p* = 0.8). The rates of sufficiency and success were similar for lytic and sclerotic lesions. In 16 of the 35 cases (44%) with a history of primary malignancy and in 15 of the 135 cases (11%) with no history of malignancy, the histopathology showed metastasis. The relationship between the absence of primary malignancy and histopathological results is summarized in Table 2.

**Table 1:** Relationship between Histopathological Groups, Material Sufficiency and Biopsy Success.

		Primary malignancy	Secondary malignancy	Inflammation/infection	Other	*p* value

Sufficiency	Sufficient	13 (72%)	26 (83%)	51 (92%)	62 (93%)	0.03
	Not sufficient	5 (28%)	5 (17%)	4 (8%)	4 (7%)	
Accuracy	True	12 (92%)	26 (100%)	48 (94%)	50 (80%)	0.006
	False	1 (8%)	0	3 (6%)	12 (20%)	
Success rate	Successful	12 (66%)	26 (83%)	48 (87%)	50 (75%)	0.1
	Fail	6 (34%)	5 (17%)	7 (13%)	16 (25%)	
Total		18	31	55	66	

**Table 2:** Relationship between the Absence of Primary Malignancy and Histopathological Results.

		Primary malignancy	Secondary malignancy	Inflammation/infection	Other	*p* value

Malignancy History	Present	0	16 (52%)	6 (11%)	13 (20%)	<0.001
	Absent	18 (100%)	15 (48%)	49 (89%)	53 (80%)	
Total		18	31	55	66	

Cutting needles were used in 167 of 170 biopsies. The Jamshidi needle was used in 137 cases, while the Tru-Cut needle was used in 30. The needles had similar results in terms of obtaining sufficient material and resulting in a successful biopsy. The most-used needles were 13 gauge (119) and 14 gauge (25), but no significant difference was found between wide-scale needles (11 and 13 gauge) and relatively thin needles (14–20 gauge) in terms of biopsy success (*p* = 0.6). In the analysis of biopsies at the thoracic, lumbar, and sacral levels, very similar rates were found in material sufficiency. Although biopsy success was higher for lumbar and sacral vertebral lesions compared to the thoracic level, this difference was not statistically significant (*p* = 0.2). The cervical level was not analyzed because of an insufficient number of biopsies. No significant difference was found between the average length of samples obtained with different needle approaches (*p* = 0.1) (Table 3). No significant difference was found between the average length of the samples when the lesions’ radiological appearances were grouped as lytic, sclerotic, or mixed type with both lytic and sclerotic areas (*p* = 0.1) (Table 3).

**Table 3:** Mean Size of Biopsy Materials with Different Needle Approaches and Different Radiological Appearances.

Needle Approach	N	Sample Length (mm) Mean ± Std. Deviation

Transcostovertebral	6	11.3 ± 6.6
Posterior	20	12.4 ± 7.3
Posterolateral	37	10.9 ± 5.9
Transpedicular	104	9.3 ± 5.6
**Radiological Appearance**	
Lytic	122	10.2 ± 6.3
Mixed	25	10.3 ± 4.7
Sclerotic	20	9.3 ± 5.7
**Total**	167	

## Discussion

Among the variables that can affect biopsy success (such as the lesion’s histopathological features), the biopsy success rates for primary bone tumors, sclerotic bone lesions, and cystic bone lesions [17,18,19,20] have rarely been reported. Kattapuram et al., Ward et al., and Fyfe et al. reported that thick needles led to higher rates of diagnostic accuracy compared to thin needles [16,21,22]. Compared to the lumbar area, there is a higher rate of success in the thoracic area [15,23,24,25]. Brugieres et al. and Lis et al. reported high rates of success (90% and 100%, respectively) for the cervical region [25,26]. Kattapuram et al., Kornblum et al., and Lis et al. reported high levels of success (86%, 92%, and 96%, respectively) for the sacral level [15,16,26]. In contrast, Ozerdemoglu et al. reported a very low success rate (12%) for the sacral area [27]. In our study and many others, no relationship was found between biopsy success and vertebral level. In our vertebrae biopsies, we did not find a significant difference in success rates with regard to the aforementioned variables. In contrast to previous studies, we analyzed the probable relationship between the radiological appearance of the lesion and the sample size and did not find any statistically significant differences. In our study, no association was found between needle type and sample size.

One of the factors affecting the accuracy of CT-guided percutaneous biopsy is the lesion’s histological type. For instance, the diagnostic accuracy rate for metastatic lesions has been reported to be higher than for primary bone tumors [16]. The highest false positive rate has been reported in benign tumors, inflammatory lesions, and pseudotumoral lesions, while the accuracy rate for malignant lesions has been reported as high [6]. The accuracy rates are lower for lesions with fibrotic, collagenous, or necrotic areas; inflammatory lesions; aneurysmal bone cysts; and hemangiomas. In infectious lesions (osteomyelitis, spondylitis), diagnosis becomes more difficult due to the nonspecificity of histological features, the inability to grow bacteria in microbiological cultures, and antibiotic treatment in chronic infection cases. In malignancies such as lymphoma or myeloma, the diagnosis can be difficult since it depends on immunohistochemical methods. In our study, the biopsy success rate was low in patients with a final diagnosis of myeloma. This may be due to insufficient material obtained during needle biopsy or the sampling of nondiagnostic tissue.

With regard to the needle approach, a posterolateral approach should not be the first choice because of the high risk of complications; however, this approach can be used in the presence of an intervertebral disc mass or a wide paravertebral mass. The transcostovertebral approach is used only in the thoracic area and is preferred when the posterolateral approach is impossible due to a long transverse process. The transpedicular technique is useful [21,28], but it can be difficult to penetrate the cortex [8]. The limitation of this technique is that it is not able to reach the intervertebral disc. Thus, transforaminodiscal biopsy becomes advantageous in this situation.

Another disadvantage of the transpedicular technique is the possibility of harm to the medial and inferior walls of the pedicle as well as injury to the spinal canal structures or nerve roots. However, we did not encounter this type of damage. The increased success rates in the sacral area are due to the greater ease of intervention and the ability to obtain more samples in the absence of vital structures, such as the spinal cord or major veins (26). Another limitation of the transpedicular approach is the diameter of the pedicle [29,30]. However, this technique is useful when the lesion is within or neighboring the pedicle.

Complication rates of 0–26 per cent have been reported for percutaneous vertebral biopsies [7]. Previous studies have reported nerve root damage, local infection, pneumothorax, vascular injury, paraspinal hematoma, temporary paresis, paraplegia, meningitis, and death [7,31,32,33]. No serious complications were seen in our study; only pain and minor hemorrhage were observed.

One limitation of our study was the small number of cases at the cervical level. In addition, this was a retrospective study, so the data on complications were available only from the patients’ files.

## Conclusion

In conclusion, CT-guided percutaneous needle biopsies are important and recurrent methods that can be performed under local anesthesia and are less invasive processes when compared with open biopsy. In this study, no difference was found between age, gender, the location of the lesion, radiological appearance and size, needle type and approach, and material sufficiency and biopsy success. In terms of the histopathology of the lesions, material sufficiency and biopsy success were found to be low in the primary malignancy group. In patients with a history of malignancy, those with a biopsy result of metastasis were high in number. No association was found between the length of the sample taken and needle approach or the radiological appearance of the lesion. CT-guided percutaneous needle biopsy is a fast, economical, and safe diagnostic tool with a high success rate and a low risk of complications. This method should therefore be used for most cases of vertebral lesions.
